# Arterioureteral Fistula in the Setting of Radical Cystoprostatectomy and Ileal Conduit Creation

**DOI:** 10.7759/cureus.24533

**Published:** 2022-04-27

**Authors:** Sahil Zaveri, Bhavin Shah, Mazhar Soufi, Zbigniew Moszczynski, Jamshed Zuberi

**Affiliations:** 1 Surgery, St. Joseph's University Medical Center, Paterson, USA; 2 Surgery, CarePoint Health Bayonne Medical Center, Bayonne, USA

**Keywords:** ileal conduit creation, prostatectomy, cystectomy, bladder cancer, hematuria, arterioureteral fistula

## Abstract

Arterioureteral fistulas (AUF) following ileal conduit reconstruction are rare and not well-studied. We present a life-threatening bleed from an AUF due to an ileal conduit urinary diversion. In addition, we identify the challenges in the diagnostic process as well as management strategies. We present a 63-year-old male with ileal conduit reconstruction for bladder cancer with an AUF developing years after the reconstruction, which was ultimately managed with angioplasty.

## Introduction

An ileal conduit (IC) is the most frequent surgical procedure performed for total urinary diversion. In this operation, a segment of the ileum is used to reconstruct a bladder for the collection of urine. It is most indicated following radical cystectomy for bladder cancer, advanced colorectal, or gynecologic malignancies. The conduit has many complications; some frequent issues are urinary insufficiency, recurrent urinary tract infections (UTIs), small bowel obstruction, or stomal stenosis. Fistulation following the procedure is relatively rare but can be life-threatening. They are primarily iatrogenic and occur due to extensive pelvic surgery, radiation, indwelling ureteral stenting, or fibrosis [1]. Hematuria is usually the earliest clinical manifestation, and angiography is the diagnostic modality of choice. Confirming a diagnosis can be challenging due to the lack of sensitivity of the study, and up to one-third of the cases are missed on initial angiography [2]. Therefore, a high index of suspicion should be maintained while working up hematuria in this group of patients.

## Case presentation

**Figure 1:**
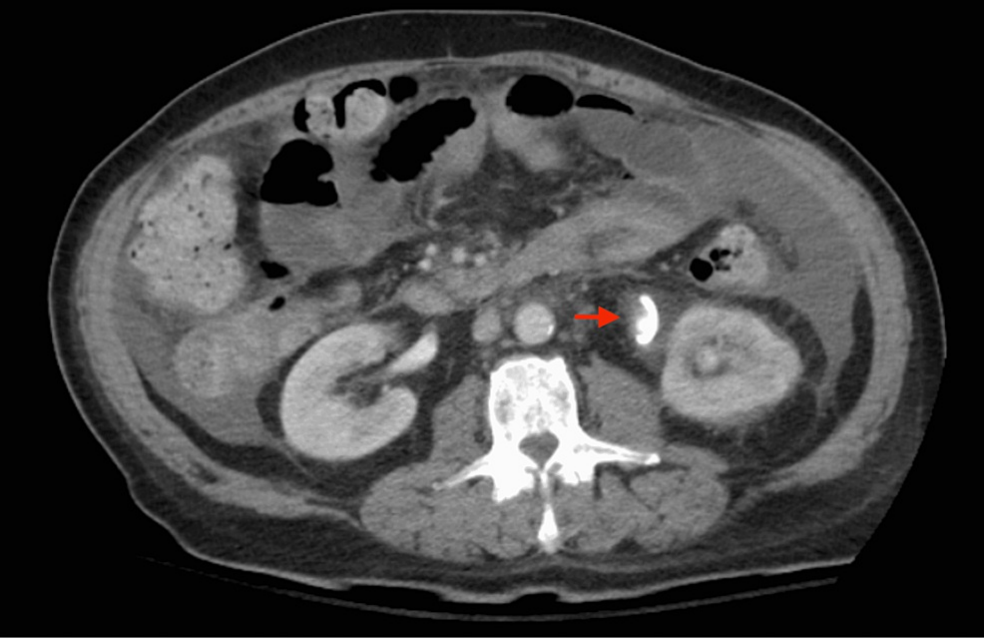
Computed tomography angiography on admission. Imaging revealed intravenous contrast in the distal portion of the ileal conduit raising concern for active extravasation.

**Figure 2:**
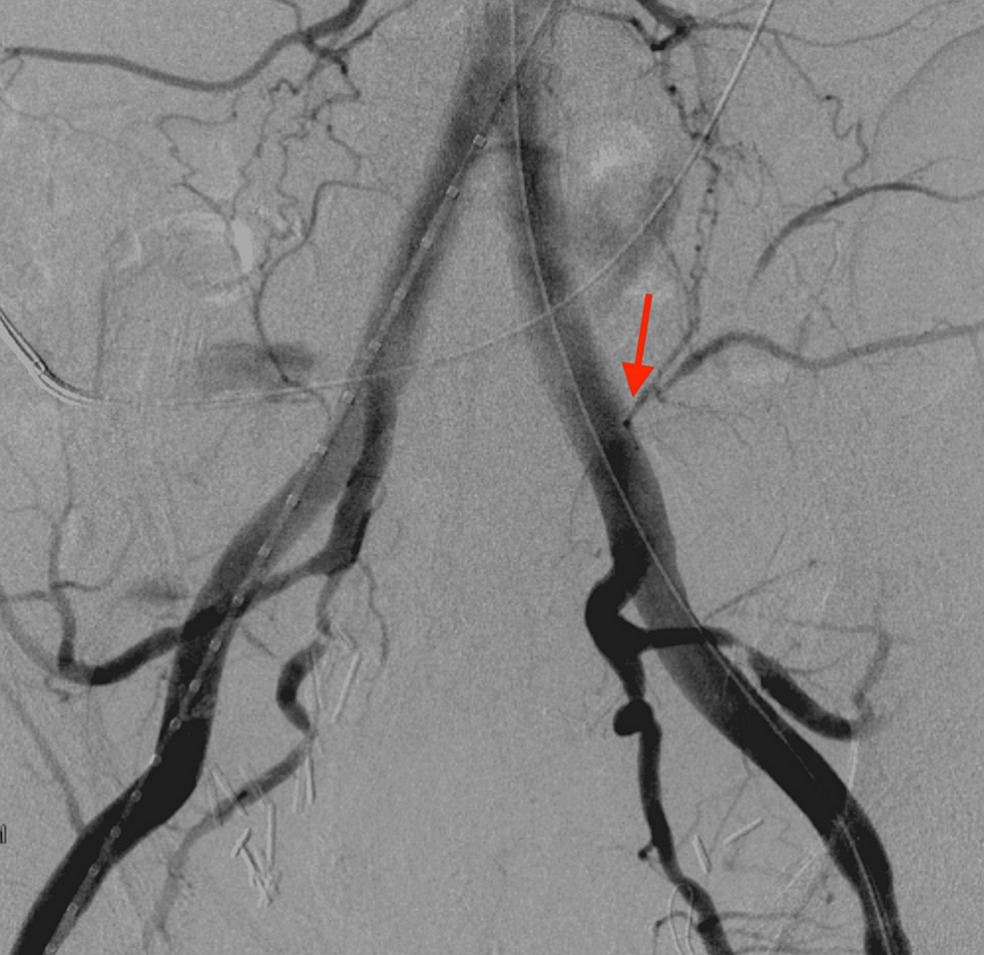
Intraoperative X-ray fluoroscopy. X-ray fluoroscopy revealed extravasation of intravenous contrast from the left common iliac to the ureter.

A 63-year-old male with a past surgical history of a radical cystoprostatectomy and an IC created four years ago for a stage 3A transitional cell carcinoma of the urinary bladder presented to the hospital. In the emergency department, 300 ml of blood drainage through the IC ostomy was observed. His IC procedure was complicated by early stricture formation around the distal ureteral anastomosis. This resulted in UTIs and required multiple balloon urethroplasties with stent placements. Work-up on presentation revealed acute blood loss anemia requiring a transfusion. However, computed tomography angiography (CTA) of the abdomen and pelvis did not disclose any active extravasation. Of note, the patient demonstrated evidence of disease progression on radiographic surveillance two months from the current presentation. Therefore, the bleeding was believed to have originated from a vascular fistula of either malignant or inflammatory etiology secondary to chronic indwelling catheterization with recurrent UTI. As a result, the patient was transferred to a referred center with advanced endovascular technology. Here, the patient underwent a ureteroscopy. Upon gaining entry to the left ureter, bright blood was encountered, thus confirming the diagnosis of AUF. On the second day of hospitalization, the patient had a repeat CTA of the abdomen and pelvis while blood was actively emanating from the ostomy of the IC. At this time, imaging revealed increased density in the distal IC following the administration of intravenous contrast raising suspicion of active extravasation (Figure 1). The patient was immediately taken to the vascular catheterization lab. The initial aortogram with bilateral iliofemoral runoff did not demonstrate contrast extravasation. However, a J stent was used to manipulate the ureteral stent away from both common iliac arteries. A repeat aortogram at this point demonstrated active extravasation from the left common iliac artery into the ureter. An iliac stent graft was deployed to bridge the bleeding point from the left common iliac artery, which was then dilated using balloon angioplasty. Post-stent deployment angiography confirmed proper placement and sealing of the arterial leak (Figure 2).

## Discussion

Arterioureteral fistulas (AUFs) are a rare and potentially fatal disease process that requires high clinical suspicion and a low threshold for intervention. The pathophysiology in our case could have been a combination of predisposing factors, such as pelvic surgery, irradiation, and an indwelling catheter. This is consistent with the findings of a multi-institutional analysis, in which 118 cases of AUF were studied. From those cases, 73.7% had chronic indwelling ureteral stents, 70.3% had malignancy, 69.5% had prior pelvic surgery, and 75% had prior radiation treatment [3]. As stated previously, hematuria is the most prominent complaint ranging from microscopic hematuria to frank hemorrhage. Possible symptoms are the lower back, flank, and abdominal pain. Other characteristics include infection, pseudoaneurysm, and hydronephrosis [2,4]. Although there is not yet confirmed pathophysiology of fistula formation, there are proposed theories. Regarding the vasculature, it is believed that any pelvic surgical intervention or radiation can disrupt the vasa vasorum making it easier for potential necrosis to occur, which can later lead to fistula formation. Regarding the ureters, similar exposures can cause ureteral devascularization or loss of elasticity, which could ultimately predispose to a fistula formation [3,4].

The last theory involves the physical proximity of the pulsatile arterial vessel to the ureter, which could cause microtraumas, especially in stented ureters [3,5]. As mentioned above, the best diagnostic study is a standard angiography. The sensitivity of the angiography can range between 23 and 41% but can be increased to 63% when the stent is manipulated and mobilized during the procedure, as was done in our patient [3,6]. Nonetheless, it is essential that a multi-imaging and multidisciplinary approach is taken. Therefore, diagnostic studies should not be limited to CT of the abdomen and pelvis, ultrasound, urography, ureterography, and angiography [6].

Just as there were various pathophysiological theories as to what caused the fistulas, there are numerous management approaches based on the anatomy. From a vascular surgery standpoint, local reconstruction, ligation with or without extra-anatomic bypass, or internal iliac ligation can be performed. From a urological perspective, the ureter can be managed through a primary repair, ligation, reimplantation, or a diversion through the nephrostomy tube which can be created [4-6]. These options are not exclusive to being done endovascularly or by open surgery. Due to the complications and risks of open surgeries being much more significant, the preference is to perform endovascular interventions. Even though there is a preference and anticipated superior outcomes, no retrospective study has established the superiority of the endovascular approach compared to open surgery [3,4].

The majority of patients who underwent endovascular interventions did not require further interventions on follow-up. One study followed eight patients with microscopic hematuria due to a fistula who had an endovascular intervention over a three-year period. Three patients had recurrent hematuria, two had repeat open surgeries, and one had a repeat endovascular procedure [7]. One study found four patients who had hematuria during endoscopic ureteral stent manipulation. This happened in our patient since the J stent was used to pull back the ureter from the iliac arteries to induce and better visualize the bleeding from the fistula [8]. Once the ureter was pulled back, bleeding from the fistula was appreciated from the left common iliac artery territory. An iliac stent graft was deployed to repair the bleeding from the left common iliac artery, which was then post dilated with balloon angioplasty.

Luckily for our patient, endovascular access was possible, and an open procedure was unnecessary, which would come with its own set of complications [9,10]. The main goal of the surgical interventions would be to control the hemorrhage by attaining vascular continuity, in this case, placing a stent. The second aim would be to ensure adequate and continuous urinary drainage [11]. Due to a high clinical suspicion, the complexity and urgency of addressing the AUF were mainly appreciated upon further diagnostic studies. In our patient, only after ureteroscopy evidence of a fistula with the pulsatile bleeding was identified as the source of gross hematuria in this patient. Both CTAs could not rule in nor out the fistula, but the probability substantially increased considering the clinical picture and the ureteroscopy. The fistula was appreciated and immediately addressed during the joined endovascular and urological approach. This echoes the findings and conclusions from the retrospective study referenced earlier [7-11].

## Conclusions

An AUF is a communication between the iliac artery and the IC. This is a rare but life-threatening complication from an IC creation. It is imperative to have a high index of suspicion in the presence of hematuria with clinical predisposing factors. Further diagnostic studies should be immediately pursued to attain clarity and confirmation of the presence of an AUF. Endovascular angioplasty can be safe and effective in managing this complication. Thus, it is paramount that immediate intervention is conducted to mitigate mortality and morbidity in a potentially fatal condition.
